# “DOST” Model to Link and Support Drug Resistant TB Patients From Private Sector: An Experience From Delhi, India

**DOI:** 10.3389/fpubh.2022.835055

**Published:** 2022-05-11

**Authors:** Vindhya Vatsyayan, Theresa Pattery, Khasim Sayyad, Jason Williams, Arnab Pal, Vikas Panibatla, Ashwani Khanna

**Affiliations:** ^1^Clinton Health Access Initiative (CHAI), New Delhi, India; ^2^Johnson & Johnson Global Public Health R&D, Beerse, Belgium; ^3^TB Alert India, Hyderabad, India; ^4^State TB Office, New Delhi, India

**Keywords:** DOST model, drug resistant tuberculosis, Delhi, India, private sector, linkages

## Abstract

**Background:**

The National TB Elimination Programme (NTEP) has quite successfully involved private sector for referral of presumptive drug resistant TB (DR-TB) patients for molecular testing and referral for DR-TB management. There was a challenge as all the referred patients were not reaching to the facilities. A “DOST” intervention model was implemented to strengthen the patient care pathway. We conducted this study to describe the patient care cascade, the clinico-demographic characteristics of patients linked to the treatment and to estimate the mean turn-around time for drug resistant TB care services.

**Methods:**

It is a cross-sectional study conducted at New Delhi during the period July 2019-December 2020 under programmatic settings.

**Results:**

A total of 9,331 patients were subjected to CB-NAAT test and 382 (4%) were found to be resistant for rifampicin and 231 (76%) were initiated on treatment in the public sector under NTEP.

**Conclusion:**

The DOST intervention model developed to link the DR-TB patients from private sector to the public sector DR-TB centers is found to be efficient and effective.

## Introduction

Tuberculosis is a major public health problem worldwide and India accounts for nearly 27% of the global burden (1). Among these TB patients, the ones who are diagnosed with drug resistant TB (DR-TB) have the worst outcomes and can transmit the infection to their household members and the community (2). In India, the estimated number of DR-TB patients is 12.4 million and the proportion of new and retreatment patients are found to be 22 and 37%, respectively (3). To address the critical gaps in DR-TB patient care, the country has developed a specific programmatic management of drug resistant tuberculosis (PMDT) guidelines for decentralized management of patients across the country (4). The major source of DR-TB notification in the country is the public sector followed by private sector. In India, the first point of contact for a presumptive TB patient is a practitioner from the private sector and the reasons toward this inclination are accessibility, trust, affordability, and feasibility (5).

The diagnosis of a DR-TB patient is currently a laboratory diagnosis performed by subjecting the presumptive patient's sputum or tissue samples to National Tuberculosis Elimination Programme (NTEP)-accredited laboratories for drug susceptibility testing. Based on the results, the patients are initiated on treatment at DR-TB centers, as per the programmatic guidelines (4). The process for diagnosing and initiating treatment for DR-TB patients should be immediate and any delay here will lead to poor treatment outcomes and spread of infection. Based on informal patient/community surveys conducted by NTEP, there seems to be clear evidence that most of the presumptive DR-TB patients were referred from the private sector and delays were incurred for accurate diagnosis and treatment initiation. The continuum of care for TB patients starts with identification of the patient in the community based on symptoms and signs and then subjected to appropriate laboratory diagnostic tests for DR-TB; if found positive, it will be followed by pre-treatment evaluation completion, and linking the patient from the private sector to the public sector DR-TB centers for observation for a week with treatment advice, counseling, and continuation of the treatment at their domicile with periodic monitoring of the patients to prevent the development of extensively drug resistant TB (XDR-TB) (4). This treatment pathway is complex and has significant number of operational challenges as it is dependent on factors like (a) patient's health seeking behavior (b) multiple diagnostic tests that need to be conducted at specialized clinical laboratories that are sparse, difficult to access and has long turn- around time for results (c) long treatment duration of up to 24 months with drugs having frequent side-effects making it a challenge to complete treatment and prevent loss to follow up. To address the challenges and strengthen the process, we developed a health systems intervention model named as “DOST” (which means “Friend” in Hindi) to effectively link and refer private DR-TB patients to DR-TB centers under the programme and provide treatment adherence support using a combination of mobile health (mHealth) Information communication and technology (ICT) solutions and trained field personnel. We conducted this study with the following objectives (1) to determine the patient care cascade for DR-TB patients diagnosed at private sector in New Delhi during July 2019-December 2020 with regards to (a) number (proportion) of DR-TB patients linked to DR-TB centers (b) number (proportion) of DR-TB patients who underwent pre-treatment evaluation and Line Probe Assay for second line TB treatment (c) number (proportion) of patients who were initiated on DST-guided regimen (2) To describe the clinico-demographic characteristics of patients linked to the treatment (3) to estimate the mean turn-around time for drug resistant TB care services.

## Materials and Methods

This is a cross-sectional study design conducted at New Delhi during the period July 2019–December 2020. The state of New Delhi has a population of 30.2 million with high literacy rate of 88% (6). Under the NTEP, the state is divided into 25 districts. In 2020, a total of 86,914 TB patients were notified of which 59,709 (68%) were from public sector and 27,205 (27%) from private sector. There are 25 district DR-TB centers and four nodal DR-TB centers to provide specialized services to DR-TB patients under the program (3). A total of 2,024 MDR/RR patients diagnosed, out of which 1,625 (80%) MDR/RR patients were initiated on treatment (3). The state has three accredited laboratories to perform second line drug susceptibility testing (SL-DST) (3). The health facilities have capacity to perform pre-treatment evaluation, follow-ups, patient management during adverse drug reactions and necessary drug supply to initiate patients on short course DR-TB regimen. The health care services are mainly delivered through the Aam Aadmi Mohalla Clinics, Dispensaries, and seed primary urban health centers, Polyclinics, Society hospitals and Super Specialty Hospitals. The city has nearly 41 public health facilities and 600 private health facilities registered under NIKSHAY (a web-based online portal from National TB Elimination Programme) for TB notification.

### Processes for DR-TB Care Services and the Role of Health System Under NTEP

The processes and functionalities of health system are as described (a) Identification of presumptive TB or DR-TB: The health facility must correctly recognize, provide counseling, and promptly refer the patient (b) sample collection: the laboratory technician or the health care personnel should provide appropriate counseling and follow the standard operating procedure (SOP) in collecting the sample (4) (c) sample transportation: an identified agency should ensure necessary logistics and adhere to SOP for transportation (d) testing and reporting: the laboratory technician should be proficient, prompt and perform correct reporting (e) Pre-treatment evaluation and initiation of treatment: the health facility and health care personnel should counsel the patient, co-ordinate and provide proper referral linkages (f) ambulatory care of DR-TB patient: the health care personnel should provide adequate counseling, proper linkages, extend support and monitoring of patients (g) regular follow-up: the health care personnel should regularly conduct clinical follow-up examination along with necessary laboratory investigations (h) treatment outcome or end of treatment: the health care personnel should provide adequate support for clinical follow-up (i) long term follow-up: the health personnel should conduct home-visits, screen family members as an active case finding strategy and test the symptomatic to diagnose TB or DR-TB (4).

### DOST Intervention Model

The structure of the model was based on three key pillars (1) the field staff called as treatment coordinators (2) Call center and (3) Mobile health Information Technology platform called as Connect for Life™ (Johnson & Johnson). The role of each of these pillars are further described below.

#### Treatment Coordinators

They provided linkage and referral support for the patients. They developed a liaison between private practitioners, patients, and public sector health facilities. Their main role was to sensitize the private physicians on the cartridge based nucleic acid amplification test (CB-NAAT) availability and the services offered by the project Joint Efforts for Elimination of Tuberculosis (JEET) which included sputum collection and transport facility and updating the results to the treating physicians (7). They co-ordinate with the JEET projects sputum collection and transport agents, the programme personnel at laboratories, health facilities and DR-TB centers to minimize the out-of-pocket expenditure to the patients, decrease multi-center health visits and reduce the turn-around time for testing.

#### Call Centers

It was established to regularly follow-up the patients diagnosed with DR-TB. The call centers initially register the patients on the mHealth Connect for Life™ platform and follow them up at regular time-intervals for disease counseling, pre-treatment evaluation, treatment adherence, health empowerment/nutrition counseling and adverse drug reactions. All the call services were customized to the convenience of patients. An alert mechanism was developed to identify the patients not responding to calls and to inform treatment coordinators for further follow-up. The center was manned by personnel trained for counseling and fluent to communicate in local languages. To ensure utmost quality of patient services, the call recordings were randomly scrutinized by technical experts.

#### Connect for Life™

The main role of this mobile health enabled platform is to benefit the patients by connecting them to health care providers for support, to complete their check-ups and treatment on a timely manner. Once the diagnosed patients are registered on treatment through coordinators on this platform, automated communication are sent in the form of text messages and voice messages to the patient's mobiles on benefits of treatment adherence along with pill and visit reminders. Such a differentiated service delivery model platform helps the treatment coordinator to focus mainly on “lost to follow-up” that is triggered by alerts in the system, thereby reducing the burden on managing all patients in a similar way. The platform helps in updating the patient treatment status to all the concerned health care providers on a real time basis. It provides a window to swiftly act in case of emerging non-adherence.

## Results

**Figure 1:**
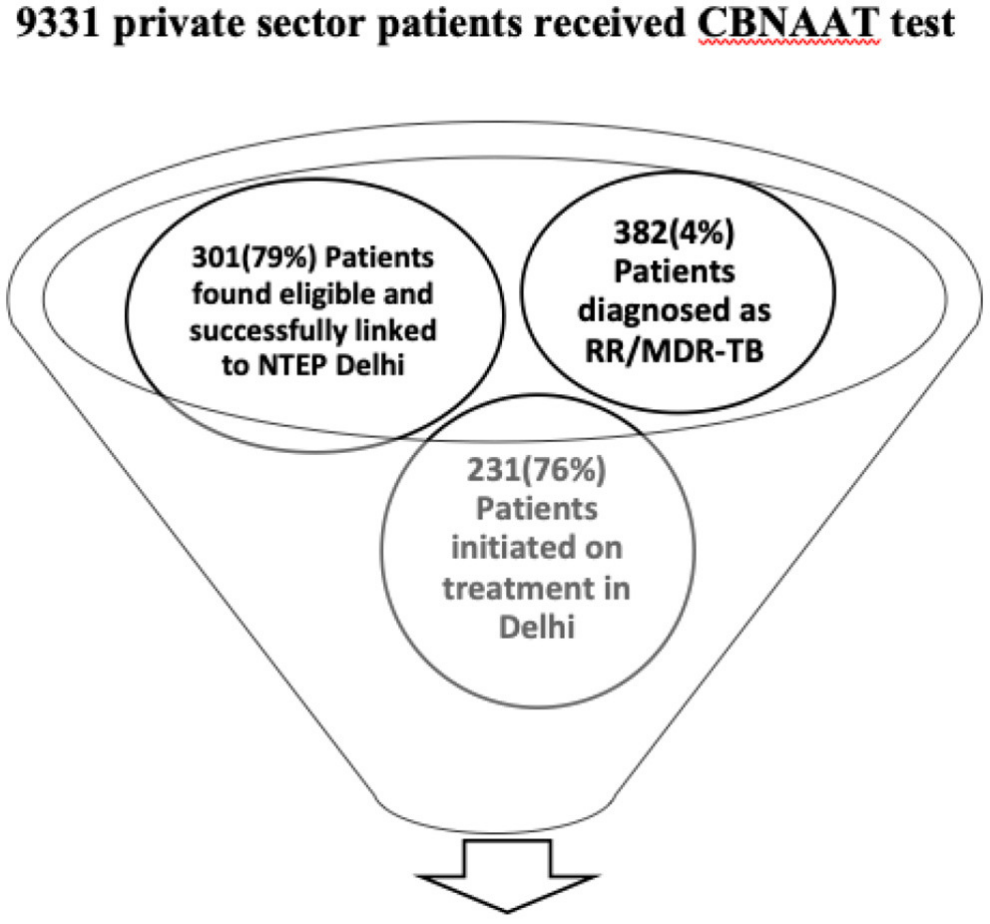
Patient care cascade of private sector patients subjected to CB-NAAT test and initiated on treatment.

In the state of Delhi during the study period, a total of 9,331 patients were subjected to CB-NAAT test and 382 (4%) were found to be resistant for rifampicin. The project had engaged 112 private health facilities and 301/382 (79%) patients diagnosed were from private sector and were referred to district DR-TB centers (see Figure 1).

### Diagnosed DR-TB Patients Care Cascade

Of the 301 diagnosed patients linked to the programme, 231 (76%) were initiated on treatment after completing pre-treatment evaluation at public health facilities. Of the 70 patients who were not initiated on treatment, 28 (9%) patients were found to be rifampicin-resistant negative on repeating CB-NAAT, 20 (7%) patients were transferred out of Delhi and initiated on treatment at their respective states, 12 (4%) patients died before treatment initiation, 10 (3%) patients opted out of the linkage programme. The reasons for opting out from the linkage from programme were refusal to take treatment because of delay in the PTE process, long wait times and delay in response from NTEP programme personnel, private practitioner not being convinced about the drugs given at public health facility, patient or the family not satisfied with the quality of services provided at the health systems.

Of the 231 patients initiated on treatment, 198 (86%) of patients were found to be eligible for second line drug susceptibility testing (SL-DST) by Line probe assay (LPA) and 134/198 (68%) had completed testing in the stipulated time. Out of the remaining 64 patients, the results were not known for 62 patients and the details of 2 patients were not available with the districts.

### Clinico-Demographic Characteristics of Patients (Table 1)

The mean age of the patients were 32 years (range 2–85) and 186 (49%) were females and 196 (51%) were males. Majority (146, 38%) of the patients were in the age group of 15–30 years and 85% of patients had pulmonary tuberculosis. Most (215, 57%) of the DR-TB patients were retreatment patients and 356 (93%) of patients were rifampicin resistant. Only 209 (64%) patients knew their HIV status and majority (86%) of them were counseled for coronavirus disease (COVID-19). Majority (183/231, 80%) of patients initiated on treatment were on shorter MDR-TB regimen. Among those patients who underwent second line LPA testing and whose results were known 54% (72/134) patients were found to be sensitive for second line injectables and fluoroquinolones.

**Table 1:** Clinico-demographic characteristics of DR-TB patients, DOST project, July 2019 to Dec 2020.

	**Sex**		
	**Female**	**Male**	**Total**
	**(*n* = 186)**	**(*n* = 196)**	**(*N* = 382)**
	**No. (%)**	**No. (%)**	**No. (%)**
**Age (years)** 0–14	72 (39)	51 (26)	123 (32)
15–30	71 (38)	75 (38)	146 (38)
31–45	21 (11)	38 (19)	59 (15)
46–60	11 (6)	25 (13)	36 (9)
> 60	11 (6)	7 (4)	18 (5)
**Type of disease**			
Extrapulmonary	36 (19)	22 (11)	58 (15)
Pulmonary	150 (81)	174 (89)	324 (85)
**Type of DR-TB patient**			
Primary case	81 (44)	84 (43)	165 (43)
Retreatment case	105 (56)	112 (57)	217 (57)
**HIV status**			
Negative	104 (65)	103 (63)	207 (64)
Not known	81 (34)	92 (36)	173 (35)
Positive	1 (1)	1 (1)	2 (1)
**COVID-19 counseling**			
No	57 (14)	66 (13)	123 (14)
Yes	129 (36)	130 (87)	259 (86)
**Type of DR-treatment regimen** ***(n** **=** **231)***			
Shorter MDR TB regimen	86 (78)	97 (84)	183 (80)
DST guided XDR regimen	12 (11)	8 (7)	20 (9)
Bedaquiline DST guided regimen	9 (8)	4 (3)	13 (6)
Delamanid DST guided regimen	2 (2)	2 (2)	4 (2)
Isoniazid monoresistance	3 (1)	8 (4)	11 (3)
**SL-LPA results** ***(n** **=** **198)***			
SLI and fluoroquinolone resistant	4 (4)	2 (2)	6 (3)
SLI and fluoroquinolone sensitive	17 (19)	36 (33)	53 (27)
SLI resistant and fluoroquinolone sensitive	1 (1)	2 (2)	3 (1)
SLI sensitive and fluoroquinolone resistant	37 (42)	35 (32)	72 (36)
Testing not completed	31 (34)	33 (31)	64 (32)

### Turn Around Time for DR-TB Care Services

#### TAT for CB-NAAT Testing

It is the time point from the identification of presumptive DR-TB patient by a private practitioner till the declaration of the patients' CB-NAAT results. The mean TAT was found to be 2.6 days during the project period and was 10.1 days before the commencement of the project.

#### TAT for Pre-treatment Evaluation

It is the time point from the diagnosed DR-TB patient to complete his pre-treatment evaluation either at public or private health facilities. The mean TAT was found to be 8 days during the project period and was 14 days before the commencement of the project.

#### TAT for Second Line LPA

It is the time point from identification of an eligible DR-TB patient for second line LPA and knowing the patients results from the laboratory. The mean TAT was found to be 27 days during the project period and was 100 days before the commencement of the project.

## Discussion

It is one of the first intervention model conducted in India under programmatic settings to facilitate and strengthen the linkages of diagnosed DR-TB patients under private sector to the programme. Our study findings reveal that three out of four DR-TB patients diagnosed from the private sector were effectively linked to the DR-TB centers and seven out of 10 patients who were eligible for SL-DST underwent line probe assay testing; while the remaining three out of 10 were EP cases and were not tested due to lack of appropriate samples.

### DOST Intervention Model

The strategy and intervention of this model was found to be effective and efficient. The continuous engagement of treatment coordinators with the private sector physicians and the diagnosis of DR-TB patients along with networking of program staff and laboratory staff was found to be fruitful. This is clearly demonstrated with evidence as the uptake of CB-NAAT tests in the private sector increased up to 3.5 times and the turn-around time for laboratory testing decreased from 10 to 2 days when compared to the time before the intervention and these are in line with the guidance provided by the programme (8). There was also a drastic reduction of multiple visits to the hospitals by the patients for various pre-treatment evaluation tests. The continuous real time monitoring of patients by the call center through reminders using social media applications like WhatsApp and text messages; making calls to patients or their caregivers at a convenient time and utilizing the opportunity to educate them regarding the importance of treatment, the process and motivating them for early treatment initiation led to gradual improvement in pre-treatment evaluation to an average of 8 days when compared to 14 days during the pre-intervention period. The average turn-around time for second line LPA also substantially reduced from 100 days to 26 days; we anticipated that it will be reduced to 14 days, but the prevailing COVID-19 pandemic was a deterrent (4). The mHealth application provided the real time status of patients who were in different stages of pathways for diagnosis, treatment initiation and treatment adherence to the program managers and health care providers; immediate remedial measures were taken if there were any deviation in the timelines from the standard pathways.

### Programmatic Implications

The study findings have the following programmatic implications. Firstly, it is found that most of the DR-TB patients belonged to the age group of 0–30 years. The ratio of male to female was found to be almost similar unlike we routinely find under the programme. This brings out the fact that the younger generation has more trust on the quality of services provided by the private sector. The program needs to adopt innovative strategies of health system strengthening and various modalities to improve the quality of care given to the patients at public healthcare facilities.

Secondly, nearly 60% of patients treated at private sector belonged to re-treatment category and around 60% of them were found to be fluoroquinolone resistant. This is a wake-up call for the programme to improve the DR-TB patient care pathway, as otherwise these patients would remain churning within the health system without timely access to correct diagnosis and treatment and in the process would have lost precious life saving time, money, and accidentally spread DR-TB infections within their household and into the community. It is urgent and critical for the programme to relook into the strategies adapted for private sector engagement especially in metropolitan and tier one cities across the country. The programme should envisage to adopt such a “DOST model” which is feasible to replicate, pragmatic, result oriented with measurable outcome indicators and lessens the burden on health systems with a differentiated service delivery model approach. With the advent of newer Programmatic Management of DR-TB guidelines (2021), it is essential to sensitize and update the private sector on the novel diagnostic and treatment services and processes available in the region under the program (4). Enrichment of private sector knowledge can be done through posters, messages, videos, calls and one-to-meetings using the tightly knitted mechanisms of treatment coordinators, call center, private health facilities and the patients.

Thirdly, it is seen that patient had to make multiple visits to health facilities for diagnosis and pre-treatment evaluation before initiation of treatment. A step closer to quality improvement is to develop a one-stop public health facility with short turn-around times. Even during country wide lockdowns, pandemic states and when patients are too sick or incapacitated to travel to the clinics, care and adequate support with health empowerment can be provided optimally on a real time basis, using mHealth solutions such as Connect for Life™ with dedicated care givers.

Finally, the COVID-19 pandemic has seen some of the exemplary multi-sectoral involvement and integration of health care delivery services in the country. Drawing lessons from the pandemic, the health machinery platform utilized for COVID-19 surveillance needs to be adapted with emphasis on identification, detection, and treatment of drug resistant TB patients.

## Conclusion

To conclude, the intervention model developed to link the DR-TB patients from private sector to the DR-TB centers is found to be efficient and effective. The programme should take bold steps in replicating such models across the country for meaningful engagement of private sector and to achieve the ambitious target of eliminating TB by 2025 in India.

## Data Availability Statement

The original contributions presented in the study are included in the article/supplementary material, further inquiries can be directed to the corresponding author.

## Ethics Statement

Ethical review and approval was deemed not necessary as it included the analysis of programmatic data that were captured in records and reports. The same was reviewed and approved by State TB Office, Delhi.

## Author Contributions

VV, TP, JW, AP, and VP: conceptualization. VV, TP, JW, and AP: methodology. VV and KS: project management. VV, VP, AK, and JW: supervision. VV and TP: data analysis. VV, KS, and TP: writing first draft of manuscript. All authors critical comments and approval of final draft of manuscript and have read and agreed to the published version of the manuscript.

## Funding

This research was funded by Johnson & Johnson Global Public Health R&D.

## Author Disclaimer

The views expressed in this article are of the authors and do not represent the views of the authors' organizations.

## Conflict of Interest

VV and AP were employed by Clinton Health Access Initiative (CHAI). TP and JW were employed by Johnson & Johnson Global Public Health R&D. KS and VP were employed by TB Alert India. The remaining author declares that the research was conducted in the absence of any commercial or financial relationships that could be construed as a potential conflict of interest.

## Publisher's Note

All claims expressed in this article are solely those of the authors and do not necessarily represent those of their affiliated organizations, or those of the publisher, the editors and the reviewers. Any product that may be evaluated in this article, or claim that may be made by its manufacturer, is not guaranteed or endorsed by the publisher.
